# Динамика показателей минерального обмена у госпитализированных пациентов с COVID-19, влияние этиотропной и патогенетической терапии

**DOI:** 10.14341/probl13304

**Published:** 2023-08-30

**Authors:** И. С. Маганева, А. С. Бондаренко, А. П. Милютина, А. Р. Елфимова, Е. Е. Бибик, Л. В. Никанкина, Н. В. Тарбаева, А. К. Еремкина, Н. Г. Мокрышева

**Affiliations:** Национальный медицинский исследовательский центр эндокринологии; Национальный медицинский исследовательский центр эндокринологии; Национальный медицинский исследовательский центр эндокринологии; Российский национальный исследовательский медицинский университет имени Н.И. Пирогова; Национальный медицинский исследовательский центр эндокринологии; Национальный медицинский исследовательский центр эндокринологии; Национальный медицинский исследовательский центр эндокринологии; Национальный медицинский исследовательский центр эндокринологии; Национальный медицинский исследовательский центр эндокринологии; Национальный медицинский исследовательский центр эндокринологии

**Keywords:** COVID-19, гиперпаратиреоз, гипокальциемия, дефицит витамина D, барицитиниб

## Abstract

ОБОСНОВАНИЕ. Высокая распространенность новой коронавирусной инфекции (COVID-19), сохраняющаяся в настоящее время, диктует необходимость детального изучения ее патогенеза. Актуальным представляется исследование взаимосвязи нарушений минерального обмена с тяжестью течения COVID-19.ЦЕЛЬ. Изучить динамику основных показателей минерального обмена у госпитализированных пациентов в активной стадии COVID-19, в том числе в зависимости от проведения этиотропной и патогенетической терапии.МАТЕРИАЛЫ И МЕТОДЫ. На базе ГНЦ РФ ФГБУ «НМИЦ эндокринологии» Минздрава России проведено исследование с включением 106 пациентов (в возрасте ≥18 лет) с клинически или лабораторно подтвержденной COVID-19. Комплексное лабораторное обследование, включая показатели минерального обмена и маркеры воспаления, инструментальная оценка тяжести COVID-19 проводились до начала патогенетической терапии, на 3, 7-е сутки госпитализации и перед выпиской. Статистический анализ выполнялся с помощью программы Statistica 13 (StatSoft, США).РЕЗУЛЬТАТЫ. В первый день госпитализации отмечалась высокая частота гипокальциемии — в 40,6% случаев по уровню альбумин-скорректированного кальция; суммарная распространенность дефицита/недостаточности витамина D составила 95,3%. При этом вторичный гиперпаратиреоз был выявлен только в 14,2% случаев. Уровень общего кальция у пациентов с SpO2<93% был достоверно ниже, чем в группе с SpO2≥93% (p=0,001), а более низкие значения общего кальция сыворотки крови сочетались с более тяжелым поражением легочной ткани по данным мультиспиральной компьютерной томографии (r=-0,26, р=0,007, поправка Бонферрони p0=0,002). При сравнительном анализе показателей минерального обмена в течение госпитализации (между 1, 3, 7-ми сутками госпитализации и перед выпиской) на фоне терапии барицитинибом было выявлено статистически значимое увеличение альбумин-скорректированного кальция к окончанию госпитализации (p<0,001, критерий Фридмана, р0=0,01). При попарном сравнении подгрупп в зависимости от получаемой терапии на 3-и сутки выявлен статистически значимо более низкий уровень альбумин-скорректированного кальция у пациентов, получающих одновременно терапию барицитинибом и тоцилизумабом по сравнению с подгруппой пациентов, находящихся на этиотропном лечении (2,16 [2,13; 2,18] ммоль/л против 2,23 [2,19; 2,28] ммоль/л, р=0,002, U-тест, р0=0,012).ЗАКЛЮЧЕНИЕ. Для пациентов с тяжелым течением коронавирусной инфекции характерны высокая распространенность дефицита/недостаточности витамина D и гипокальциемии. Выявлены ассоциации между показателями кальция и тяжестью поражения легочной ткани, что характеризует гипокальциемию как важный предиктор тяжелого течения и неблагоприятного исхода при COVID-19. Патогенетическая терапия барицитинибом, в том числе в комбинации с тоцилизумабом, способствует достижению нормокальциемии, однако это требует подтверждения в дальнейших исследованиях.

## Abstract (en)

The high prevalence of COVID-19 requires the research progress on the disease pathogenesis. There is a lot of data confirming the association between mineral metabolism and the severity of COVID-19.AIM: To study the dynamics of mineral metabolism parameters in patients with a confirmed COVID-19 at the time of hospitalization and after discharge, including the impact of etiotropic and pathogenetic therapy on them.MATERIALS AND METHODS: A single-center study of 106 patients (aged ≥18 years) with clinically or laboratory confirmed diagnosis of COVID-19 was carried out at the Endocrinology Research Centre, Moscow. Baseline biochemical parameters, including serum calcium, phosphorus, albumin, 25(OH)D, parathyroid hormone (PTH), inflammatory markers, and instrumental assessment of COVID-19 severity were performed before specific immunotherapy, as well as on 3rd and 7th days of hospitalization and before discharge. Statistical analysis was performed with Statistica 13 software (StatSoft, USA).RESULTS: On the first day, hypocalcemia (low albumin-adjusted calcium level) was detected in 40.6% of cases, the prevalence of vitamin D deficiency/insufficiency amounted to 95.3% of cases. At the same time, secondary hyperparathyroidism was identified only in 14.2% of patients. A comparative analysis of mineral metabolism during hospitalization (between 1, 3, 7 days of hospitalization and before discharge) during baricitinib treatment revealed a statistically significant increase in albumin-adjusted calcium by the end of hospitalization (p<0.001, Friedman criterion, Bonferroni correction p0=0.01). A pairwise comparison of subgroups, depending on the therapy, revealed a statistically significantly lower level of albumin-adjusted calcium on 3rd day among patients on baricitinib monotherapy or combined with tocilizumab compared with a subgroup of patients undergoing etiotropic treatment (2.16 [2.13; 2.18] mmol/l vs 2.23 [2.19; 2.28] mmol/l, p=0.002, U-test, Bonferroni correction p0=0.012).CONCLUSION: Patients with severe coronavirus infection are characterized by a high prevalence of vitamin D deficiency and hypocalcemia. Associations between calcium and saturation as well as the severity of lung lesion characterizes hypocalcemia as an important predictor of severe course and poor outcome in COVID-19. Pathogenetic therapy with baricitinib, including in combination with tocilizumab, contributes to achieve normocalcemia, but further studies are required.

## ОБОСНОВАНИЕ

В декабре 2019 г. в китайской провинции Ухань впервые было зарегистрировано заболевание, вызванное коронавирусом SARS-CoV-2, на 80% филогенетически сходным с вирусом SARS-CoV и на 50% с вирусом MERS-CoV [1]. SARS-CoV-2 стал причиной беспрецедентной по своим масштабам и последствиям пандемии новой коронавирусной инфекции (COVID-19). По данным Университета Джонса Хопкинса, на конец марта 2023 г. в мире подтверждено более 676 млн случаев заболевания с более 6,8 млн смертей [2]. Учитывая, что COVID-19 продолжает непрерывно распространяться во всем мире, изучение патогенеза заболевания, поиск факторов, влияющих на его течение, формирование новых стратегий лечения не теряют своей актуальности.

В литературе имеются многочисленные данные о связи инфекционных заболеваний, в том числе COVID-19, с нарушениями минерального обмена. Гипокальциемия описана как отличительная особенность при данном заболевании: согласно данным метаанализа J.W. Martha и соавт., ее распространенность составляет в среднем 55% (от 23 до 87%) [3]. Кроме того, гипокальциемия является независимым фактором риска госпитализации и неблагоприятного исхода заболевания [4]. Анализ показателей минерального обмена также позволяет выявить высокую распространенность гипофосфатемии (29,7%) [5], в то время как данные о сывороточной концентрации магния противоречивы. Среди стационарных больных регистрировалась как гипо- (32–48%), так и гипермагниемия (9,6–14%), при этом последняя была выше среди пациентов в отделениях интенсивной терапии [6]. Дефицит 25(OH)D обнаруживается по меньшей мере у 50% пациентов [7]. Интересно, что для пациентов с COVID-19 на фоне вышеописанных нарушений характерно развитие функционального гипопаратиреоза (до 40% случаев) [8].

## ЦЕЛЬ ИССЛЕДОВАНИЯ

Целью нашей работы было изучение динамики основных показателей минерального обмена у госпитализированных пациентов в активной стадии COVID-19, в том числе в зависимости от проведения этиотропной и патогенетической терапии.

## МАТЕРИАЛЫ И МЕТОДЫ

## Объем исследования

Проведено одноцентровое интервенционное динамическое сравнительное нерандомизированное исследование, в которое были включены пациенты с COVID-19, прошедшие лечение в Центре COVID-19 на базе ГНЦ РФ ФГБУ «НМИЦ эндокринологии» Минздрава России в мае-июне 2020 г.

## Критерии постановки диагноза

Диагноз COVID-19 устанавливался согласно Временным методическим рекомендациям Министерства здравоохранения Российской Федерации «Профилактика, диагностика и лечение новой коронавирусной инфекции (версия 5, COVID-19)» [9].

Диагноз сахарного диабета (CД) устанавливался при уровне HbA1c ≥6,5 % и глюкозы плазмы натощак ≥7,0 ммоль/л и/или глюкозы крови при поступлении ≥11,1 ммоль/л [10].

## Критерии включения и исключения

Критерии включения: подтвержденная клинически или лабораторно новая коронавирусная инфекция; подписанное информированное согласие.

Критерии исключения: наличие ранее диагностированных хронического гипопаратиреоза, первичного гиперпаратиреоза (ПГПТ); сахарного диабета (СД); морбидного ожирения (ИМТ более 40 кг/м²); снижения скорости клубочковой фильтрации (СКФ) менее 60 мл/мин/1,73 м², бронхиальной астмы в стадии обострения; прием препаратов, влияющих на кальциевый обмен (препараты кальция, витамина D, бисфосфонаты, деносумаб, терипаратид, диуретики), наличие психических заболеваний, беременности.

## Дизайн исследования

В 1-й день госпитализации у всех пациентов была выполнена клиническая оценка тяжести COVID-19 (термометрия, определение насыщения крови кислородом (сатурация, SpO2)), лабораторная (общеклинический анализ крови с оценкой лейкоцитарной формулы, биохимический анализ крови (С-реактивный белок (СРБ), ферритин, лактатдегидрогеназа (ЛДГ), креатинин, кальций общий, альбумин, фосфор, магний, глюкоза, гликированный гемоглобин (HbA1c)), гормональный анализ крови (интактный паратиреоидный гормон (иПТГ), кальцитонин), маркеры воспаления и иммунного ответа (прокальцитонин, интерлейкины-6 и -1бета (IL-6, IL-1β), фактор роста фибробластов 23 (FGF-23), иммуноглобулин G (IgG)), а также коагулограмма, D-димер и инструментальная диагностика (оценка степени специфического поражения легких по данным мультиспиральной компьютерной томографии — МСКТ)).

Дополнительно были сформированы подгруппы пациентов в зависимости от SpO2 (подгруппа А1 — SpO2≥93%, n=74, подгруппа А2 — SpO2<93%, n=32), степени поражения легочной ткани по данным МСКТ (подгруппа Б1 — КТ1–2, n=56, подгруппа Б2 — КТ3–4, n=50), уровней СРБ (подгруппа В1 — СРБ<10 мг/л, n=10, подгруппа В2 — СРБ≥10, n=96) и IL-6 (подгруппа Г1 — IL-6<10, n=39, подгруппа Г2 — IL-6≥10, n=47).

В ходе госпитализации все пациенты получали этиотропное, патогенетическое и симптоматическое лечение согласно Временным методическим рекомендациям по профилактике, диагностике и лечению новой коронавирусной инфекции (версия 5) [9]. Также всем пациентам для профилактики тромбообразования назначалась антикоагулянтная терапия эноксапарином натрия. Этиотропная терапия (один из препаратов: гидроксихлорохин, умифеновир, аминодигидрофталазиндион натрия, препараты интерферонов, иммуноглобулин человека G, лопинавир+ритонавир) и патогенетическая терапия (тоцилизумаб, сарилумаб, барицитиниб, глюкокортикостероиды) назначались лечащим врачом после получения результатов обследования. На 3, 7-е сутки госпитализации, а также перед выпиской была проведена динамическая оценка показателей минерального обмена с последующим исследованием влияния на него терапии. Терапию барицитинибом получали 28 пациентов, однако на одном из визитов по техническим причинам анализы были недоступны, в связи с чем сравнение показателей минерального обмена в динамике проведено у 27 больных. Препараты кальция и витамина D пациентам не назначались.

## МЕТОДЫ

## Лабораторные методы исследования

Клинический анализ крови выполнялся на анализаторе Sysmex XN-1000 (Sysmex Corp., Япония): референсные интервалы (РИ) для лейкоцитов — 3,9–10х10⁹ кл./л, лимфоцитов — 0,86–4,06х10⁹ кл./л, тромбоцитов — 148–339х10⁹ кл./л; скорость оседания эритроцитов (СОЭ) — на анализаторе Test1 (Ali Fax): РИ для женщин — 2–20 мм/ч, для мужчин — 2–15 мм/ч. Биохимические исследования сыворотки крови: кальций общий (РИ 2,15–2,55 ммоль/л), альбумин (РИ 34–48 г/л для женщин, 35–50 г/л для мужчин), фосфор (РИ 0,74–1,52 ммоль/л), магний (РИ 0,7–1,05 ммоль/л), креатинин (РИ 50–98 мкмоль/л для женщин, 63–110 мкмоль/л для мужчин), глюкоза (РИ 3,1–6,1 ммоль/л), ферритин (РИ для женщин — 15–160 нг/мл, для мужчин — 30–300 нг/мл), СРБ (РИ 0,1–5 мг/л), ЛДГ (РИ 125–220 Ед/л) выполняли на биохимическом анализаторе ARCHITECH с8000 (Abbott, CША). Для исключения ложнозаниженных и ложнозавышенных показателей кальция крови производили перерасчет его концентрации с поправкой на уровень альбумина крови [11].

Определение иПТГ крови (РИ 15–65 пг/мл) проводилось на электрохемилюминесцентном анализаторе Cobas 6000 (Roche, Германия), 25(ОН)D (РИ 30–100 нг/мл) и кальцитонина (РИ 0–4,8 пг/мл для женщин, 0–11,8 пг/мл для мужчин) — на анализаторе Liaison XL (DiaSorin, Италия). СКФ определяли с учетом возраста пациента и уровня креатинина сыворотки крови по формуле CKD-EPI 2009 (рСКФ).

HbA1c (РИ 4−6%) определялся методом высокоэффективной жидкостной хроматографии на анализаторе D10 (BioRad, США).

Показатели коагулограммы определяли на анализаторе ACL TOP LAS 700 (Instrumentation Laboratory, США): РИ для АЧТВ — 28–40 с, для МНО — 0,8–1,3. Количественный высокочувствительный анализ D-димера плазмы (РИ 0–500 нг/мл) выполнен на аппарате ACL TOP 700 (IL, США). Определение IL-6 (РИ 0–10 пг/мл) осуществляли с помощью иммуноферментных наборов «Вектор-Бест» (Россия), ИЛ-1β (РИ 0–5 пг/мл) — методом иммуноферментного анализа (ELISA) при помощи стандартного набора Invitrogen, USA; прокальцитонина (РИ 0–0,05 нг/мл) — методом иммунохемилюминесценции на анализаторе Arсhitect i2000 (Abbott); IgG (РИ 5,52–16,31 г/л) — методом иммунотурбидиметрии, биохимический анализатор Arсhitect c8000 (Abbott); FGF-23 (РИ 0.1–20 пмоль/л) — методом иммуноферментного анализа (ELISA), Biomedica, Словакия.

## Инструментальные методы исследования

Индекс массы тела (ИМТ) рассчитывали по формуле: ИМТ=масса тела (кг)/рост (м²). Оценка степени насыщения крови кислородом (SpO2) проведена с помощью пульсоксиметра CS Medica MD300C2. МСКТ органов грудной полости проводилась на компьютерном томографе GE Optima CT660 (США). Протокол МСКТ формировался согласно временным методическим рекомендациям по профилактике, диагностике и лечению новой коронавирусной инфекции (версия 5) [9].

## Статистический анализ

Размер выборок предварительно не рассчитывался. Статистический анализ данных выполнен с помощью пакета прикладных программ Statistica 13 (StatSoft, США) и языка программирования для статистической обработки данных R 4.2.2. Описательная статистика количественных признаков представлена медианами, первым и третьим квартилями в виде Ме [Q1; Q3], качественных — в виде абсолютных (n) и относительных (%) частот. Сравнение двух независимых подгрупп пациентов для количественных данных выполнялось с помощью критерия Манна–Уитни (U-тест). Частоты бинарных признаков сравнивались между собой с помощью критерия Хи-квадрат Пирсона (χ²), при значении хотя бы одной ожидаемой частоты менее 5 применялась поправка Йейтса, при наличии нулевых частот использовался двусторонний точный критерий Фишера. Сравнение двух исследуемых зависимых групп (до и после медикаментозного лечения) для количественных данных выполнялось при помощи критерия Вилкоксона (W-тест), для качественных — при помощи критерия Мак-Немара. Для сравнения количественных признаков более чем в двух взаимосвязанных группах применялся критерий Фридмана, попарно данные группы сравнивались с помощью критерия Краскела–Уоллиса с последующим проведением post-hoc анализа. Корреляционный анализ проводился с помощью метода ранговой корреляции Спирмена. Исходный критический уровень статистической значимости (p) при проверке статистических гипотез принят равным 0,05. При множественных сравнениях применялась поправка Бонферрони (р0) путем коррекции критического уровня значимости. Рассчитанные уровни значимости в диапазоне от критического до 0,05 описаны в качестве индикаторов статистической тенденции.

## Этическая экспертиза

Протокол научного исследования был рассмотрен и одобрен на заседании локального этического комитета ГНЦ РФ ФГБУ «НМИЦ эндокринологии» Минздрава России от 30 апреля 2020 г. (протокол №6). Все пациенты подписали информированные согласия на участие в исследовании.

## РЕЗУЛЬТАТЫ

В исследование включены 106 пациентов (из них 63 мужчины и 43 женщины, соотношение м:ж=1,5:1). Медиана возраста составила 55 [ 45; 66] лет. При этом мужчины и женщины были сопоставимы по возрасту (p=0,031, U-тест). Продолжительность госпитализации составила 12 [ 10; 14] дней. Среди прогностически неблагоприятных факторов тяжелого течения коронавирусной инфекции оценивались ИМТ (медиана 28,0 [ 25,0; 31,2] кг/м²) и фильтрационная функция почек (рСКФ — 87,5 [ 72; 100] мл/мин/1,73 м²).

## Общее состояние пациентов при поступлении

Диагноз коронавирусной инфекции лабораторно был подтвержден у 74 пациентов, что составило 69,8%. В то же время специфическое поражение легких разной степени тяжести по данным МСКТ было выявлено в 100% случаев: 1-й степени у 13 пациентов (12,3%), 2-й — у 43 пациентов (40,6%), 3-й — у 46  (43,4%) и 4-й степени — у 4 пациентов (3,8%). Снижение SpO2 менее 93% определялось у 32 пациентов (30,2%), повышение СРБ ≥60 мг/л — у 53 (50%), IL-6 ≥10 пг/мл — у 47 обследуемых из 86 (54,7%), ЛДГ≥220 Ед/л — у 88 (83%) больных. Тяжелое течение заболевания с развитием «цитокинового шторма» было диагностировано у 30 больных (28,3%). Уровень ферритина был статистически значимо выше у мужчин (p<0,001, U-тест), в остальном значимых различий в маркерах воспалительного процесса по полу выявлено не было (табл. 1).

**Table:** Таблица 1. Характеристика маркеров воспалительного процесса у пациентов с COVID-19 при поступлении в стационар Примечание. Поправка Бонферрони p0=0,004.

Признак	РИ	Все пациенты	Мужчины	Женщины	р, U-тест
n	Ме [ Q1; Q3]	n	Ме [ Q1; Q3]	n	Ме [ Q1; Q3]
Лейкоциты, х10⁹ кл./л	3,9–10,0	106	5,93 [ 4,48; 8,11]	63	6,71 [ 4,86; 8,97]	43	5,28 [ 4,00; 6,82]	0,027
Лимфоциты, х10⁹ кл./л	0,86–4,06	106	1,06 [ 0,75; 1,5]	63	1,08 [ 0,78; 1,57]	43	1,05 [ 0,73; 1,36]	0,328
Тромбоциты, х10⁹ кл./л	148–339	106	201 [ 161; 254]	63	185 [ 149; 234]	43	207 [ 171; 285]	0,016
СОЭ, мм/ч	для женщин: 2–20 для мужчин: 2–15	106	32,5 [ 22; 47]	63	29 [ 18; 39]	43	37 [ 22; 54]	0,008
ЛДГ, Ед/л	125–220	106	287 [ 233; 374]	63	315 [ 233; 413]	43	267 [ 230; 349]	0,136
Ферритин, нг/мл	для женщин: 15–160 для мужчин: 30–300	106	431,9 [ 211,3; 867,4]	63	801,2 [ 329,4; 1017,5]	43	230,6 [ 154,9; 452,5]	<0,001
СРБ, мг/л	0,1–5	106	59,45 [ 25,8; 112,4]	63	66,0 [ 22,0; 133,4]	43	51,3 [ 30,0; 79,5]	0,401
IL-6, пг/мл	0–10	86	12,70 [ 4,64; 40,10]	54	16,20 [ 4,64; 50,40]	32	12,24 [ 4,66; 29,805]	0,453
IgG, г/л	5,52–16,31	38	11,685 [ 9,91; 12,65]	22	10,66 [ 8,90; 12,50]	16	12,485 [ 10,995; 13,05]	0,040
FGF-23, пмоль/л	0,1–20	105	0 [ 0; 0]	62	0 [ 0; 0]	43	0 [ 0; 0]	0,398
IL-1β, пг/мл	< 5	17	18,90 [ 6,61; 68]	12	13,55 [ 2,18; 86,10]	5	20,5 [ 15; 68]	0,423
D-димер, нг/мл	0–500	100	233,5 [ 153,5; 383]	62	227,5 [ 146; 396]	38	240,5 [ 160; 349]	0,709

## Характеристика показателей минерального обмена при поступлении

Медиана общего кальция составила 2,20 [ 2,12; 2,29] ммоль/л, альбумина — 41 [ 38; 44] г/л, альбумин-скорректированного кальция 2,18 [ 2,11; 2,24] ммоль/л. Гипокальциемия по уровню альбумин-скорректированного кальция определялась в 40,6% наблюдений (n=43). Случаев гиперкальциемии в исследуемой группе выявлено не было. Медиана 25(ОН)D была 12,50 [ 7,83; 17,50] нг/мл, при этом дефицит определялся в 81,1% случаев (n=86), недостаточность — в 14,2% (n=15), оптимальный уровень — всего в 4,7% (n=5). Медиана иПТГ составила 39,65 [ 29,09; 56,84] пг/мл, а вторичный гиперпаратиреоз (ВГПТ) был диагностирован в 14,2% случаев (n=15). В то же время низкий уровень иПТГ в 1-е сутки госпитализации отмечался в 5,7% наблюдений (n=6). Уровень фосфора был определен у 97 больных, из них у 9 (9,3%) выявлена гипофосфатемия, а у 10 (10,3%) — гиперфосфатемия. Одинаково часто встречалась как гипер-, так и гипомагниемия (в 1% случаев).

В подгруппе А2 (SpO2<93%) были определены статистически значимо более низкий уровень общего кальция сыворотки крови (2,14 [ 2,08; 2,22] ммоль/л против 2,23 [ 2,14; 2,32] ммоль/л, p=0,001, U-тест, p0=0,002) и тенденция к более низким показателям альбумин-скорректированного кальция (2,13 [ 2,08; 2,20] ммоль/л против 2,19 [ 2,12; 2,25] ммоль/л, р=0,023, U-тест, p0=0,002).

При сравнении подгрупп Б1 (КТ1–2) и Б2 (КТ3–4), во второй также были выявлены значимо более низкие уровни общего кальция крови (2,15 [ 2,10; 2,23] ммоль/л против 2,26 [ 2,16; 2,34] ммоль/л, р=0,001, U-тест, p0=0,002), а различия по уровню альбумин-скорректированного кальция не достигли статистической значимости (2,17 [ 2,11; 2,25] ммоль/л против 2,19 [ 2,11; 2,24] ммоль/л, р=0,959, U-тест).

Более низкие показатели общего кальция сыворотки сочетались с более тяжелым поражением легочной ткани (r=-0,26, р=0,007, метод Спирмена, р0=0,001). Выявлены умеренные отрицательные корреляции с тенденцией к статистически значимой между уровнем фосфора с СОЭ (r=-0,25, p=0,012, р0=0,001), с IL-6 (r=-0,24, р=0,034, р0=0,001) и с D-димером (r=-0,29, р=0,005, р0=0,001).

У пациентов с гипокальциемией чаще развивался цитокиновый шторм (53% против 32,2%, р=0,005, χ²), и чаще требовался перевод в реанимацию (61,3% против 35,4%, р=0,007, χ²). Случаев летального исхода зафиксировано не было.

## Сравнительный анализ показателей минерального обмена в ходе госпитализации

При оценке динамики показателей минерального обмена в ходе госпитализации было отмечено, что уровень иПТГ статистически значимо увеличивался на 3-и сутки и далее снижался к моменту выписки, оставаясь при этом в пределах референсного интервала. Среди пациентов, у кого при госпитализации был выявлен низкий уровень иПТГ, уже на 3-и сутки он нормализовался. Минимальные значения альбумин-скорректированного кальция отмечались в 1-е сутки. Уже с 3-го дня фиксировалось его повышение, и данная динамика сохранялась до конца госпитализации. Похожая ситуация была отмечена для фосфора и магния (рис. 1).

**Figure:**
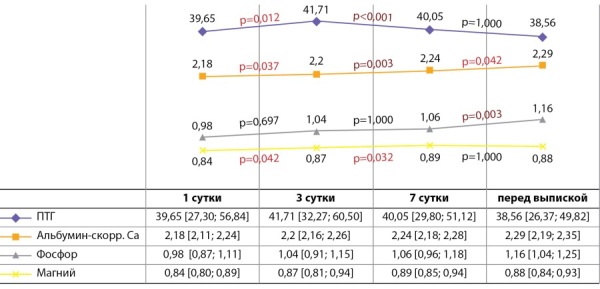
Рисунок 1. Динамика показателей минерального обмена в ходе госпитализации.Примечание. На рисунке представлены динамические изменения медиан показателей минерального обмена, в таблице — Ме [ Q1; Q3]; группы сравнивались с помощью критерия Краскелла–Уоллиса с последующим проведением post-hoc анализа. Поправка Бонферрони р0=0,010.

Перед выпиской нормокальциемия была достигнута в 84% случаев против 59,4% в день поступления (p<0,001), и, соответственно, уменьшилась частота гипокальциемии (по альбумин-скорректированному кальцию) (16% против 40,6%, p<0,001, при p0=0,002). Частота ВГПТ перед выпиской снизилась до 11,3%, однако различия не достигли статистической значимости (рис. 2).

**Figure:**
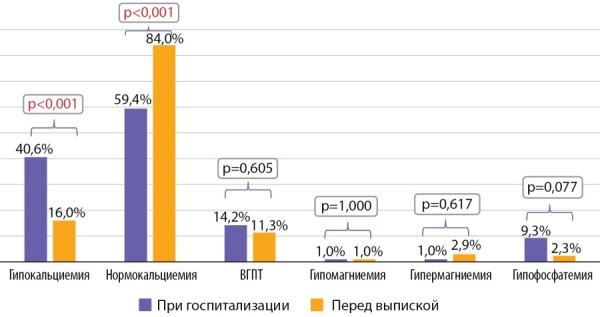
Рисунок 2. Нарушения минерального обмена у пациентов при госпитализации и перед выпиской.Примечание. Значение р рассчитано с использованием критерия Мак-Немара. Поправка Бонферрони p0=0,002.

## Взаимосвязь показателей фосфорно-кальциевого обмена и проводимой терапии COVID-19

Этиотропную терапию получали 37,7% пациентов (n=40), из них 7,5% (n=3) — лопинавир+ритонавир. Патогенетическую терапию назначали в 62,3% (n=66), спектр назначаемых препаратов и их комбинаций представлен на рис. 3.

**Figure:**
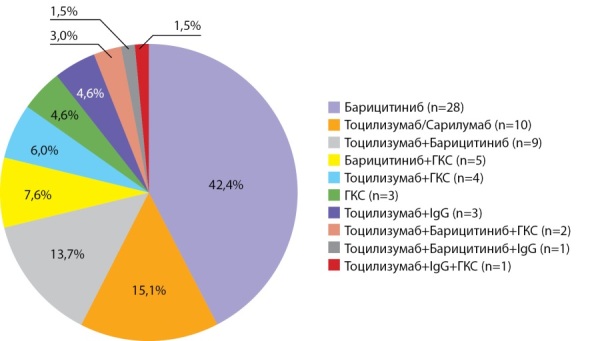
Рисунок 3. Спектр препаратов патогенетической терапии COVID-19, получаемой участниками исследования.Примечание. ГКС — глюкокортикостероиды, IgG — иммуноглобулин человеческий G.

Подгруппы были сопоставимы по показателям минерального обмена в день поступления (табл. 2).

**Table:** Таблица 2. Сравнительный анализ показателей минерального обмена в подгруппах, в зависимости от получаемой терапии, до ее назначения Примечание. Поправка Бонферрони p0=0,007.

Показатель	Патогенетическая терапия	Этиотропная терапия	р, U-test Me [ Q1; Q3]
n	Me [ Q1; Q3]	n	Me [ Q1; Q3]
ПТГ, пг/мл	66	39,65 [ 29,09; 56,84]	40	39,88 [ 28,11; 56,14]	0,834
25(ОН)D, нг/мл	66	13,65 [ 9,13; 18,40]	40	10,5 [ 6,0; 15,9]	0,067
Кальций общий, ммоль/л	66	2,17 [ 2,12; 2,26]	40	2,26 [ 2,12; 2,32]	0,049
Альбумин-скорректированный кальций, ммоль/л	66	2,16 [ 2,10; 2,23]	40	2,21 [ 2,12; 2,28]	0,074
Фосфор, ммоль/л	60	0,97 [ 0,86; 1,15]	37	1,03 [ 0,94; 1,14]	0,243
Магний, ммоль/л	64	0,83 [ 0,795; 0,88]	38	0,85 [ 0,81; 0,90]	0,304
FGF-23, пмоль/л	42	0,760 [ 0,550; 1,732]	20	1,048 [ 0,591; 2,002]	0,541

При оценке частоты основных нарушений фосфорно-кальциевого обмена гипокальциемия несколько чаще встречались в подгруппе пациентов, которые в последующем получали патогенетическую терапию, но статистическая значимость не была достигнута (45,5% против 33,3%, р=0,222, χ²).

На фоне монотерапии барицитинибом при анализе показателей минерального обмена при поступлении, на 3, 7 дни госпитализации и перед выпиской нами было выявлено статистически значимое повышение как общего, так и альбумин-скорректированного кальция, а также фосфора и магния к моменту выписки (табл. 3).

**Table:** Таблица 3. Динамика показателей минерального обмена на фоне терапии барицитинибом Примечание. Поправка Бонферрони p0=0,010.

Признак	1 сутки	3 сутки	7 сутки	Перед выпиской	p, критерий Фридмана Me [ Q1; Q3]
n	Me[ Q1; Q3]	n	Me[ Q1; Q3]	n	Me[ Q1; Q3]	n	Me[ Q1; Q3]
ПТГ, пг/мл	27	39,40[ 29,09; 62,01]	27	41,45[ 37,4; 65,4]	27	41,36[ 30,18; 51,12]	27	39,38[ 25,90; 48,00]	0,084
Кальций общий, ммоль/л	27	2,15[ 2,1; 2,25]	27	2,16[ 2,14; 2,24]	27	2,19[ 2,14; 2,29]	27	2,27[ 2,16; 2,38]	0,004
Альбумин-скорректированный кальций, ммоль/л	27	2,15[ 2,11; 2,19]	27	2,19[ 2,17; 2,24]	27	2,24[ 2,18; 2,30]	27	2,29[ 2,21; 2,37]	<0,001
Фосфор, ммоль/л	23	0,88[ 0,79; 0,99]	23	0,95[ 0,91; 1,21]	23	0,97[ 0,89; 1,10]	23	1,17[ 1,03; 1,24]	<0,001
Магний, ммоль/л	26	0,85[ 0,82; 0,89]	26	0,90[ 0,83; 0,96]	26	0,92[ 0,89; 0,98]	26	0,89[ 0,87; 0,95]	0,001

При этом при попарном сравнении визитов, статистически значимая положительная динамика отмечалась уже на 7 сутки по сравнению с первым днем пребывания в стационаре (2,23 [ 2,18; 2,30] ммоль/л против 2,15 [ 2,11; 2,19] ммоль/л, p<0,001, W-тест).

На фоне терапии тоцилизумабом/сарилумабом отмечалась лишь тенденция к статистически значимому повышению альбумин-скорректированного кальция ко дню выписки (р=0,042, критерий Фридмана, при p0=0,010).

При сочетанной терапии барицитинибом и тоцилизумабом уровень альбумин-скорректированного кальция также повышался, изменения достигли статистической значимости ко дню выписки (р=0,010, критерий Фридмана, p0=0,010). Различий в показателях минерального обмена по остальным терапевтическим комбинациям нами выявлено не было.

Учитывая вышеизложенные результаты, проведен сравнительный анализ показателей минерального обмена между 4 подгруппами в зависимости от получаемой терапии: 1 подгруппа получала терапию барицитинибом (n=28), 2 — тоцилизумаб/сарилумаб (n=10), 3 — одновременную терапию барицитинибом и тоцилизумабом (n=9) и 4 — этиотропное лечение (n=40).

На 3 сутки госпитализации более низкий уровень альбумин-скорректированного кальция отмечался в подгруппе, получавшей комбинированную терапию барицитинибом и тоцилизумабом (p=0,014, критерий Краскела-Уоллиса, p0=0,012). При попарном сравнении подгрупп в зависимости от получаемой терапии, на 3 сутки подтвержден статистически значимо более низкий уровень альбумин-скорректированного кальция в 3 подгруппе по сравнению с 4 подгруппой (2,16 [ 2,13; 2,18] ммоль/л против 2,23 [ 2,19; 2,28] ммоль/л, р=0,002, U-тест, p0=0,012). На 7 сутки отмечалась тенденция к статистически значимо более высокому уровню магния в подгруппе 1 по сравнению с подгруппой 4 (0,92 [ 0,89; 0,97] ммоль/л против 0,88 [ 0,82; 0,94] ммоль/л, р=0,015, p0=0,012).

## ОБСУЖДЕНИЕ

## Репрезентативность выборок

В период работы Центра COVID-19 на базе ГНЦ ФГБУ «НМИЦ эндокринологии» пациенты направлялись по скорой медицинской помощи с близлежащих районов Москвы, что могло исказить репрезентативность выборки для общей популяции. Нельзя точно сказать, с каким конкретно штаммом коронавируса госпитализировались пациенты. В период формирования выборок в Москве преобладали штаммы, близкие к европейской разновидности коронавируса. Однако в столицу попали и другие варианты возбудителя, в том числе из Азии.

## Сопоставление с другими публикациями

По результатам нашей работы гипокальциемия по уровню альбумин-скорректированного кальция была выявлена в 40,6% случаев, при этом ее тяжесть была ассоциирована со степенью поражения легочной ткани, что согласуется с ранее опубликованными данными [3]. В настоящий момент существует несколько гипотез, объясняющих патофизиологические механизмы развития гипокальциемии при COVID-19. Кальций используется вирусом для инвазии в клетки, экспрессии своих генов, посттрансляционного процессинга вирусных белков, а также играет важную роль в формировании структуры и высвобождении вирионов [12]. Также не исключается дополнительный вклад дефицита витамина D в развитие гипокальциемии [8].

Среди наших пациентов нецелевой уровень 25(ОН)D был определен в 95,3%, в то время как по результатам эпидемиологических исследований частота дефицита/недостаточности на территории Российской Федерации в среднем составляет 84% [13]. В группе пациентов, получающих колекальциферол (100 000 МЕ в неделю) на 7 сутки госпитализации выявлены более высокие значения нейтрофилов и лимфоцитов (p=0,047; p=0,025), а также более низкий уровень СРБ (p=0,028). Описанные факты подчеркивают значимость проведения в дальнейшем проспективных интервенционных исследований для уточнения эффективности приема препаратов кальция и витамина D в качестве дополнительных мер профилактики и лечения коронавирусной инфекции.

Примечательно, что при такой высокой частоте гипокальциемии и дефицита витамина D, ВГПТ был обнаружен всего у 14,2% больных. Результаты ранее опубликованных исследований также показывают высокую распространенность дефицита витамина D (67,9%), гипокальциемии (по ионизированному кальцию в 70,5% случаев) и при этом низкую встречаемость ВГПТ (20,5%) среди пациентов с COVID-19 [14]. Это говорит о других возможных причинах повышения иПТГ при COVID-19. А одним из возможных объяснений отсутствия ожидаемого ответа паратиреоцитов на гипокальциемию в данном случае может быть именно ее быстрое развитие на фоне острого воспалительного процесса в начале заболевания с последующей самостоятельной нормализацией уровня кальция крови по мере выздоровления. С другой стороны, в ряде работ среди пациентов с COVID-19 наблюдалось развитие обратного состояния — гипопаратиреоза. В 40% случаев при умеренной и тяжелой гипокальциемии сывороточный ПТГ был низким или низко-нормальным [8]. Причина такого неадекватного ответа околощитовидных желез остается до конца непонятной. В нашем исследовании гипопаратиреоз был выявлен в 5,7% случаев в 1 сутки госпитализации и в дальнейшем не подтвердился. Одним из предположений, объясняющих развитие транзиторного, или «функционального» гипопаратиреоза у пациентов с COVID-19, является нарушение экспрессии и синтеза гена кальций-чувствительного рецептора (CaSR) под влиянием провоспалительных цитокинов [15]. Также известно, что магний может влиять на синтез и секрецию ПТГ [16]. Хотя в работе S. Hashemipour с соавт. не выявили ассоциации между гипомагниемией и степенью гипокальциемии, а также не подтвердили зависимость гипопаратиреоза от гипомагниемии [8]. Однозначно сделать вывод о влиянии гипомагниемии на развитие функционального гипопаратиреоза в случае COVID-19 не представляется возможным. Сложность заключается также в несоответствии сывороточного уровня магния его внутриклеточному содержанию [16]. По результатам нашего исследования гипо- и гипермагниемия были выявлены с одинаковой частотой (1%) и не были связаны с низким уровнем иПТГ.

Нами выявлена прямая статистически значимая взаимосвязь между гипофосфатемией и уровнем воспалительных маркеров, в т.ч. IL-6. В литературе имеются схожие данные [17] и предлагается использовать сывороточный уровень фосфора для оценки тяжести течения заболевания, а также в качестве предиктора неблагоприятного исхода, хотя патогенез нарушений метаболизма фосфора остается не до конца ясным.

На протяжении госпитализации нами была выявлена положительная динамика в виде нормализации уровня кальция, а также тенденции к повышению фосфора и магния к моменту выписки. Похожее исследование было проведено S. Hashemipour и соавт. (n=123): к 4–6-му дню госпитализации было отмечено снижение уровня иПТГ 42,17±27,20 пг/мл до 31,28±23,42 пг/мл (р<0,001). При этом в 55% случаев степень снижения уровня кальция прогрессировала (8,32±0,52 мг/дл (2,08±0,13 ммоль/л) до 8,02±0,55 мг/дл (2,0±0,14 ммоль/л), р<0,001). Снижение уровней альбумина и иПТГ были независимыми значимыми факторами гипокальциемии и ее прогрессирования (ОШ 0,27, 95% ДИ: 1,10–1,46, р=0,001 на каждый 1 г/л снижения альбумина и ОШ 1,29, 95% ДИ 1,03–1,62, р=0,026 на каждые 10 пг/мл снижения иПТГ) [18].

В исследовании 2022 г. A. Minasi (n=111) проведена оценка частоты назначения той или иной терапии при COVID-19 (гидроксихлорохин, противовирусные препараты (лопинавир/ритонавир или дарунавир/ритонавир) и тоцилизумаб), которая составила 90,7%, 73,1% и 19,6% назначений, соответственно. При сравнении трех подгрупп, в зависимости от уровня кальция (1 подгруппа — кальций общий менее 2,02 ммоль/л (n=46), 2 — в диапазоне 2,02–2,15 ммоль/л (n=32), 3 — более 2,15 ммоль/л (n=33)) различий по частоте применения данных препаратов в ходе госпитализации коллегами выявлено не было (р=0,152, р=0,572 и р=0,378, соответственно) [19].

## Клиническая значимость результатов

По результатам нашего исследования терапия препаратом из группы ингибиторов янус-киназы — барицитинибом — оказывала значимое положительное влияние на показатели минерального обмена. В том числе, на 7 сутки госпитализации отмечено статистически значимое увеличение альбумин-скорректированного уровня кальция по сравнению с 1 днем госпитализации (p<0,001, W-тест). Похожая ситуация была выявлена при одновременной терапии барицитинибом и тоцилизумабом: уровень альбумин-скорректированного кальция статистически значимо повышался ко дню выписки (р=0,010, критерий Фридмана, при p0=0,010). При попарном сравнении данной подгруппы пациентами, получавшими этиотропное лечение, у первых на 3 сутки подтвержден статистически значимо более низкий уровень альбумин-скорректированного кальция (р=0,002, U-тест, при р0=0,012). В настоящее время до конца не ясен механизм воздействия патогенетической терапии при COVID-19 на фосфорно-кальциевый обмен. Известно, что барицитиниб ингибирует адаптор-ассоциированные протеин-киназы 1-го типа и 2-го типа (ААК-1, ААК-2), препятствуя эндоцитозу вирусных частиц и, соответственно, использованию вирусом ионов кальция для инвазии в клетку. Однако данная гипотеза требует подтверждения в дальнейших исследованиях [20].

Хотя нами не было получено достоверных данных об изменении показателей фосфорно-кальциевого обмена на фоне лечения другими препаратами, это, вероятно, могло быть связано с небольшим объемом исследуемых групп. Часть используемых в нашем исследовании препаратов (в частности, лопинавир+ритонавир) в настоящее время не применяется при лечении COVID-19, так как не доказали свою эффективность. Терапия блокаторами янус-киназ, а также генно-инженерными биологическими препаратами и ГКС не теряет своей актуальности и входит в современные алгоритмы патогенетического лечения коронавирусной инфекции. В дальнейшем требуется проведение дополнительных исследований с расширением объема выборки и оценки современной терапии на минеральный обмен.

## Ограничения исследования

Наиболее значимым ограничением является относительно небольшая мощность выборки, малые размеры отдельных групп пациентов. Стоит отметить, что в большинстве своем, в исследовании участвовали пациенты со среднетяжелым и тяжелым течением COVID-19, так как легкое течение заболевания, по большей части, не требовало госпитализации. Активное включение в исследование пациентов с легким течением коронавирусной инфекции в будущих работах может усилить значимость полученных результатов настоящего исследования. Также важно, что диагноз коронавирусной инфекции по результатам ПЦР-исследования был подтвержден только у 69,8% пациентов, хотя специфическое поражение легочной ткани было выявлено у 100% пациентов. Расхождение данных, вероятно, является следствием несовершенства использовавшихся тест-систем.

## Направления дальнейших исследований

Целесообразными будут расширение объема выборок, оценка влияния современной медикаментозной терапии на минеральный обмен, проведение сравнительного анализа нарушений минерального обмена у пациентов с вирусными инфекциями и пневмониями другой этиологии.

## ЗАКЛЮЧЕНИЕ

На сегодняшний день вклад компонентов минерального обмена в патогенез новой коронавирусной инфекции уже не вызывает сомнений. Суммарная распространенность дефицита/недостаточности витамина D в нашем исследовании превысила общепопуляционную и составила 95,3% случаев. Высокая частота гипокальциемии (40,6%), а также выявленные ассоциации уровня кальция крови с тяжестью течения заболевания (сатурацией, поражением легочной ткани по данным МСКТ) подтверждают важность определения кальциемии у пациентов с COVID-19, особенно в первые дни болезни. Более того, низкий уровень альбумин-скорректированного кальция был зафиксирован у пациентов, которым в последующем была назначена патогенетическая терапия, что характеризует гипокальциемию как важный предиктор тяжелого течения и неблагоприятного исхода при COVID-19. Интересной находкой стало положительное влияние патогенетической терапии барицитинибом и его сочетания с тоцилизумабом на нормализацию кальциемии, однако это требует изучения в дальнейших работах.

## ДОПОЛНИТЕЛЬНАЯ ИНФОРМАЦИЯ

Источник финансирования. Государственное задание №123021300171-7 «Хронический послеоперационный и нехирургический гипопаратиреоз: предикторы осложнений заболевания, контроль диагностики, лечения и мониторинга пациентов с использованием систем поддержки принятия врачебных решений» (2023–2025 гг.).

Конфликт интересов. Авторы декларируют отсутствие явных и потенциальных конфликтов интересов, связанных с публикацией настоящей статьи.

Участие авторов. Маганева И.С. — разработка концепции и дизайна исследования, проведение обследования участникам исследования, сбор материала, анализ данных, написание текста статьи; Бондаренко А.С. — анализ данных и литературы, написание текста статьи; Милютина А.П.— обработка материала, статистический анализ данных; Елфимова А.Р. — обработка материала, статистический анализ данных; Бибик Е.Е. — разработка концепции исследования, анализ данных, редактирование текста статьи; Еремкина А.К. — разработка концепции и дизайна исследования, анализ данных, редактирование текста статьи; Никанкина Л.В. — лабораторная диагностика, анализ и интерпретация данных, редактирование текста статьи; Тарбаева Н.В. — инструментальная диагностика, анализ и интерпретация данных, редактирование текста статьи; Мокрышева Н.Г. — разработка концепции и дизайна исследования, анализ данных, редактирование текста статьи.

Все авторы одобрили финальную версию статьи перед публикацией, выразили согласие нести ответственность за все аспекты публикации, подразумевающей надлежащее изучение и решение вопросов, связанных с точностью или добросовестностью любой части работы.

Благодарности. Выражается благодарность всем пациентам, участвовавшим в данном исследовании, а также всем сотрудникам ГНЦ РФ ФГБУ «НМИЦ эндокринологии Минздрава России, участвовавшим в лечении и обследовании пациентов, а также коллегам из отделения патологии околощитовидных желез и нарушений минерального обмена, которые оказывали неоценимую поддержку на всех этапах данной работы.
